# Thrombolysis in Myocardial Infarction (TIMI) Risk Score Assessment for Complications in Acute Anterior Wall ST Elevation Myocardial Infarction

**DOI:** 10.7759/cureus.8646

**Published:** 2020-06-15

**Authors:** Rozi Khan, Muhammad Samsoor Zarak, Ussama Munir, Khursheed Ahmed, Asad Ullah

**Affiliations:** 1 Internal Medicine, MedStar Union Memorial Hospital, Baltimore, USA; 2 Internal Medicine, Bolan University of Medical and Health Sciences, Quetta, PAK; 3 Internal Medicine, Bolan Medical College, Quetta, PAK; 4 Cardiology, Bahawal Victoria Hospital, Bahawalpur, PAK; 5 Cardiology, Punjab Institute of Cardiology, Lahore, PAK

**Keywords:** st-elevation myocardial infarction, diabetes mellitus, thrombolytic therapy, cardiac complications, timi score

## Abstract

Introduction and objective

Effective risk stratification is integral to the management of acute coronary syndromes. The Thrombolysis in Myocardial Infarction (TIMI) risk score for ST-segment elevation myocardial infarction (STEMI) is based on eight high-risk parameters that can be used at the bedside for risk stratification of patients presenting with STEMI. This study was designed to determine the frequency of cardiac complications of anterior wall STEMI assessed on TIMI risk score and to compare the rate of cardiac complications according to the TIMI score.

Materials and methods

An observational case series study was conducted in the Department of Cardiology at Sandeman Provincial Hospital in Quetta, Pakistan. The study duration was six months, from September 22, 2016 to March 23, 2017. A total of 369 patients were selected who had anterior wall myocardial infarction and received thrombolytic therapy, according to the inclusion and exclusion criteria. The TIMI score was calculated by proforma at the time of admission. Patients were divided into three groups: low-risk, moderate-risk, and high-risk TIMI groups. The frequency of complications of anterior wall myocardial infarction at the time of discharge was compared among these groups.

Results

The study included 285 male patients (77.2%) and 84 (22.8%) female patients. A total of 174 (47.2%) patients were smokers, 79 (21.4%) were obese, and 93 (25.2%) had hyperlipidemia. Of the 369 patients, 205 (55.6%) were included in the low-risk group, 150 (40.7%) in the moderate-risk group, and 14 (3.8%) in the high-risk group. Post-myocardial infarction arrhythmias were noted in 33 (16.09%) patients in the low-risk group and six (4%) patients in the moderate-risk group. Left ventricular dysfunction was noted in 158 (77.07%) patients in the low-risk group, 78 (52%) patients in the moderate-risk group, and seven (50%) patients in the high-risk group. Cardiogenic shock occurred in seven (3.41%) patients in the low-risk group, 47 (31.33%) patients in the moderate-risk group, and 0 (0%) patient in the high-risk group. Death occurred in seven (3.41%) patients in the low-risk group, 19 (12.66%) patients in the moderate-risk group, and seven (50%) patients in the high-risk group.

Conclusion

TIMI scoring provides a better assessment in terms of complications caused by STEMI. The complications include the mechanical and electrophysiology of the heart.

## Introduction

Coronary artery disease (CAD) is the leading cause of death worldwide. Myocardial infarction (MI) is considered one of the leading presentations of CAD. CAD is responsible for 30% of all mortalities [1]. An estimated three million people experience ST-segment elevation myocardial infarction (STEMI) per annum globally, and the average incidence of non-ST-segment elevation myocardial infarction (NSTEMI) is estimated at four million per annum [2]. STEMI is observed more frequently in men than women [3].

Ischemic heart disease is a growing cause of death in the developing countries. The population of Southeast Asia are reported to have the highest risks of CAD. In Pakistan, it is estimated that one of every five middle-aged adults may have an underlying subtle CAD. Moreover, women are at greater risk than men [4].

Evidence from several randomized clinical trials during the past two decades has established that immediate and complete restoration of flow in the occluded artery decreases infarct size, preserves left ventricular (LV) function, and improves survival rates [5,6]. Complications may occur immediately following the MI or may need time to develop.

The Thrombolysis in Myocardial Infarction (TIMI) risk score is a simple integer score based on eight prognostic parameters used at the bedside to assess the degree of underlying risk associated with STEMI [7]. It is calculated based on each risk associated with the patient and then scored from zero to 14 [8]. The risk scoring defines the prognosis in terms of mortality in the first 30 days. It is a valid means of mortality forecasting in patients with STEMI who undergo fibrinolysis [9]. There are multiple studies to calculate mortality in anterior wall STEMI with respect to the TIMI score. This study is being conducted to assess the frequency of complications of anterior wall STEMI, such as arrhythmia, LV dysfunction, cardiogenic shock, and death with respect to the TIMI score and improve management according to the risk.

## Materials and methods

This study was a descriptive, observational case series study conducted in the Department of Cardiology at Sandeman Provincial Hospital in Quetta, Pakistan. The study duration was six months, from September 22, 2016 to March 23, 2017, and included 369 patients. The sampling technique consisted of non-probability, consecutive sampling.

Inclusion criteria included all patients with anterior wall MI presenting within 24 hours after symptom onset and admitted in the critical care unit after thrombolysis, age 20-80 years, of both sexes.

Exclusion criteria included patients with a known case of chronic kidney disease and chronic liver disease, patients not willing for study, patients having a previous history of MI, and patients with acute anterior wall MI who were not candidates for thrombolytic therapy (presenting after 24 hours from onset of symptoms).

Permission was received from the ethical review committee of Bolan Medical College, Quetta, Balochistan. The consent was taken in written form from the respondents. A total of 369 patients were selected who had anterior wall MI and received thrombolytic therapy in the critical care unit of Sandeman Provincial Hospital according to inclusion and exclusion criteria. The TIMI score was calculated by proforma at the time of admission. Patients having a TIMI score of 0 to 4 were categorized as low risk, a score of 5-9 as medium risk, and a score higher than 9 as high risk.

The frequency of complications of anterior wall MI at the time of discharge was compared among these groups. All data were entered and analyzed by using Statistical Package for the Social Sciences (SPSS) version 20.0 (Armonk, NY: IBM Corp.). Age of patients and time to symptom onset were presented as mean ± standard deviation. Gender, cardiac complications, and categorization of TIMI scores were presented as frequencies and percentages. Smoking, obesity, and hyperlipidemia were other effect modifiers. Effect modifiers such as age, gender, smoking, hyperlipidemia, and categorization of TIMI scores were controlled through stratification. Post-stratification chi-square was applied by taking p < 0.05 as significant.

## Results

A total of 369 patients were included in the study. The mean age of patients was 54.36 ± 14.21 years. The largest proportion of participants were middle-aged (n = 180; 48.8%) as shown in Table 1.

**Table 1:** Distribution of patients according to age (n = 369) SD, standard deviation

Age (years)	Total	
	No. of patients	Percentage
Young (20-39)	38	10.3
Middle age (40-59)	180	48.8
Elderly (60-80)	151	40.9
Total	369	100
Mean ± SD = 54.36 ± 14.21 years		

The study included 285 (77.2%) male patients and 84 female patients (23%). A total of 174 (47.2%) patients were smokers, 79 (21.4%) patients were obese, and 93 (25.2%) patients had hyperlipidemia (Table 2).

**Table 2:** Distribution of patients with status of other variables

Confounding variables		Frequency	Percentage
Smoking	Yes	174	47.2
	No	195	52.8
Obesity	Yes	79	21.4
	No	290	78.6
Hyperlipidemia	Yes	93	25.2
	No	276	74.8

Out of 369 patients, 205 (55.6%) were included in the low-risk group, 150 (40.7%) in the moderate-risk group, and 14 (3.8%) in the high-risk group (Table 3).

**Table 3:** Thrombolysis in Myocardial Infarction risk groups

Risk	Score	No. of patients	Percentage
Low risk	0-4	205	55.6%
Moderate risk	5-8	150	40.7%
High risk	9-14	14	3.8%

Among the low-risk group, 32 (15.60%) were young, 154 (75.12%) were middle age, and 19 (9.26%) were elderly. The moderate-risk group had six patients (4%) who were young, 26 (17.33%) who were middle age, and 118 (78.66%) who were elderly. The high-risk group had 14 (100%) elderly patients (Table 4).

**Table 4:** Thrombolysis in Myocardial Infarction risk groups according to the age group

	Young, N (%)	Middle age, N (%)	Elderly, N (%)
Low-risk group	32 (15.60)	154 (75.12)	19 (9.26)
Moderate-risk group	6 (4)	26 (17.33)	118 (78.66)
High-risk group	0 (0)	0 (0)	14 (100)

In the low-risk group, 25 (12.19%) were female patients and 185 (90.24%) were male patients; in the moderate-risk group, 52 (34.66%) were female patients and 98 (65.33%) were male patients; in the high-risk group, seven (50%) were female patients and seven (50%) were male patients (Table 5).

**Table 5:** Thrombolysis in Myocardial Infarction risk groups according to gender

	Female, N (%)	Male, N (%)
Low-risk group	25 (12.19)	180 (88.24)
Moderate-risk group	52 (34.66)	98 (65.33)
High-risk group	7 (50)	7 (50)

Post-MI arrhythmias were noted in 53 (25.85%) patients in the low-risk group, six (4%) patients in the moderate-risk group, and six (42.85%) patients in the high-risk group. LV dysfunction was noted in 185 (90.24%) patients in the low-risk group, 106 (70.66%) patients in the moderate-risk group, and seven (50%) patients in the high-risk group. Cardiogenic shock occurred in 28 (13.65%) patients in the low-risk group, 52 (34.66%) patients in the moderate-risk group, and eight (57.14%) patients in the high-risk group. Death occurred in seven (3.41%) patients in the low-risk group, 19 (12.66%) patients in the moderate-risk group, and seven (50%) patients in the high-risk group (Table 6).

**Table 6:** Frequency of post-myocardial infarction complications according to Thrombolysis in Myocardial Infarction risk groups LV, left ventricular

Complications	Low risk, N (%)	Moderate risk, N (%)	High risk, N (%)	P-value
Arrhythmias	53 (25.85)	6 (4)	6 (42.85)	0.001
LV dysfunction	185 (90.24)	106 (70.66)	7 (50)	0.001
Cardiogenic shock	28 (13.65)	52 (34.66)	8 (57.14)	0.001
Death	7 (3.41)	19 (12.66)	7 (50)	0.001

By applying the Pearson chi-square test, the frequency of post-MI arrhythmias significantly correlated with TIMI risk groups (p = 0.001). Similarly, the frequency of post-MI LV dysfunction significantly correlated with TIMI risk groups (p = 0.001). The frequency of post-MI cardiogenic shock also significantly correlated with TIMI risk groups (p = 0.001). Frequency of post-MI deaths also showed a significant correlation with the risk groups (p = 0.001) (Table 6).

## Discussion

There are several validated scoring systems available for risk stratification in patients who present with STEMI. However, many such systems are not convenient because they require the integration of many variables to predict the outcomes associated with STEMI [10-12]. Some tools require assessing the severity of illness in terms of acute physiology and use the chronic health evaluation II scoring system [13]. Other scoring systems are limited to the opinion of experts and relevant investigations [14]. Some risk stratification models yielded better predictions; however, their operation relies upon complex algorithms. However, the TIMI risk assessment model is a convenient tool that can be used for early predictions based on the presenting symptoms of patients at the time of arrival to the hospital. TIMI does not use complicated algorithms, rather it is based on a simple and efficient scoring mechanism [15]. Morrow et al. found the predictive capacity of this risk score stable over multiple time points, in men and women, and in smokers and nonsmokers in the Treatment of Infarcting Myocardium Early II (TIME II) trial population in whom it was developed. Furthermore, all of the variables included in this model were independent predictors of 30-day mortality [16]. The risk score, however, showed a poor discriminative ability among nearly 50,000 elderly patients (older than 65 years) in the Cooperative Cardiovascular Project (CCP) database [17]. Subsequently, the TIMI risk index was tested as a predictor of in-hospital mortality in more than 150,000 patients with STEMI from the National Registry of Myocardial Infarction (NRMI)-3 and NRMI-4 databases [18].

Our study determined the reliability of TIMI scoring in terms of in-patient morbidity and mortality among thrombolysis-eligible STEMI patients. The mean age of this study population was 54.36 ± 14.21 years, with 151 (41.9%) patients older than 60 years, including both with and without a history of diabetes, hypertension, and smoking. The score performed well in predicting mortality, as well as morbidity in terms of post-MI arrhythmias, LV dysfunction, cardiogenic shock, and death [9,19].

There are several limitations to the study. The results of the study are significant; however, larger cohorts are required in local settings to assess the applicability of the TIMI score. The study does not include cardiac biomarkers in the study.

## Conclusions

Our study focused on the implication of TIMI scoring in order to assess the risk stratification of patients with STEMI. TIMI is a convenient tool that requires clinical data at the time of presentation to the hospital. It does not use any complex or time-consuming algorithms to assess the outcomes associated with the patient. TIMI scoring also provides a better assessment in terms of mechanical and electrophysiological complications. Therefore, it is prognostic imperative and its results need to be applied on population-based cohorts to assess the reliability of the results.
